# Novel pyrazole inhibitors for discrimination between receptor-operated and store-operated Ca^2+^ entry

**DOI:** 10.1186/1471-2210-11-S2-A18

**Published:** 2011-09-05

**Authors:** Hannes Schleifer, Regina Oppenrieder, Sonia Stürmer, Bernhard Doleschal, Michael Poteser, Toma N Glasnov, C Oliver Kappe, Klaus Groschner

**Affiliations:** 1 Institute for Pharmaceutical Sciences, Pharmacology & Toxicology, University of Graz, 8010 Graz, Austria; 2 Institute of Chemistry, Christian Doppler Laboratory for Microwave Chemistry, University of Graz, 8010 Graz, Austria

## Background

Calcium governs a wide range of cellular processes. Specifically, control of gene transcription involves Ca^2+^ entry channels that are activated by either voltage, second messengers or depletion of intracellular stores. The family of classical transient receptor potential channels (TRPC) has been implicated in both the receptor/second messenger as well as in store-operated Ca^2+^ entry pathway, and represents an attractive target for therapeutic intervention.

## Methods

We tested a series of pyrazol compound structurally related to Pyr3 [1], a recently discovered TRPC3 inhibitor, for effects on receptor- as well as store-operated Ca^2+^ entry into RBL-2H3 mast cells and HEK293 cells overexpressing TRPC3.

## Results and conclusions

We identified novel Ca^2+^ entry inhibitors, which are able to discriminate between the two tightly related pathways of receptor/second messenger-activated and store-operated calcium entry. These compounds appear suitable for selective modulation of Ca^2+^-dependent gene transcription in a variety of mammalian cells.
